# Supplementary material for: A highly effective and self-transmissible CRISPR antimicrobial for elimination of target plasmids without antibiotic selection
Source: PeerJ. 2021 Sep 6;9:e11996. doi: 10.7717/peerj.11996 (PMC8428258; doi:10.7717/peerj.11996)
Supplement: Supplemental Information 1 [file peerj-09-11996-s001.docx]

Supplementary

**A highly effective and self-transmissible CRISPR antimicrobial for elimination of target plasmids without antibiotic selection**


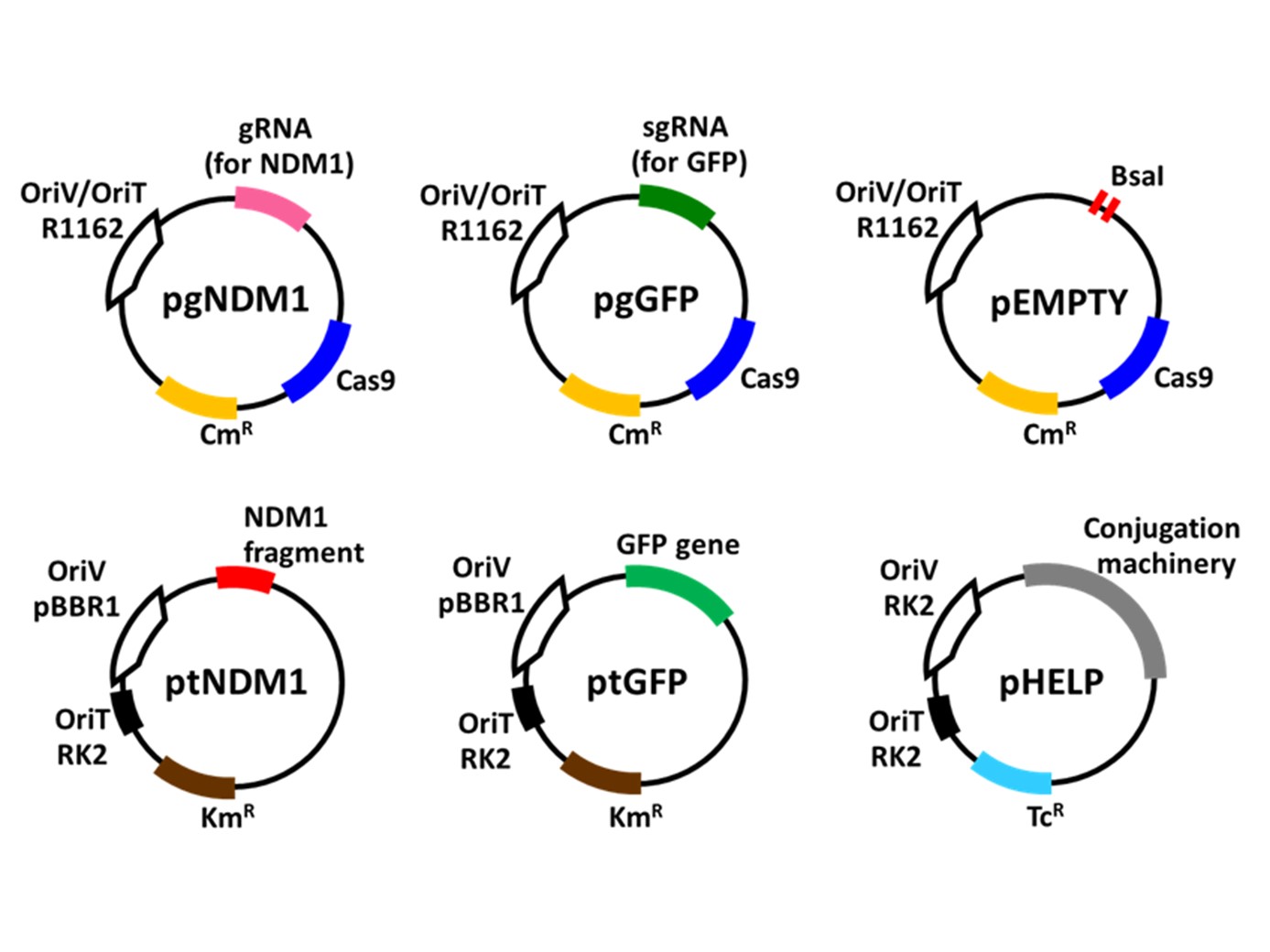


**Figure S1 The components of all plasmids used in this study.** pgNDM1 comprised a R1162 origin of replication and origin of transfer (OriV/OriT), cas9 gene, chloramphenicol resistance gene trans activating CRISPR RNA (tracrRNA) gene and CRISPR locus targeting NDM1 fragment. pgGFP and pEMPTY were similar to pgNDM1 but had CRISPR locus replaced with single guide RNA (sgRNA) targeting GFP and ‘empty’ sgRNA without a spacer sequence (only BsaI sites were present), respectively. ptNDM1 and ptGFP consisted of a pBBR1 origin of replication, RK2 origin of transfer, kanamycin resistance gene and NDM1 fragment or GFP gene, respectively. pHELP had an RK2 origin of replication and origin of transfer, tetracycline resistance gene and conjugation machinery.


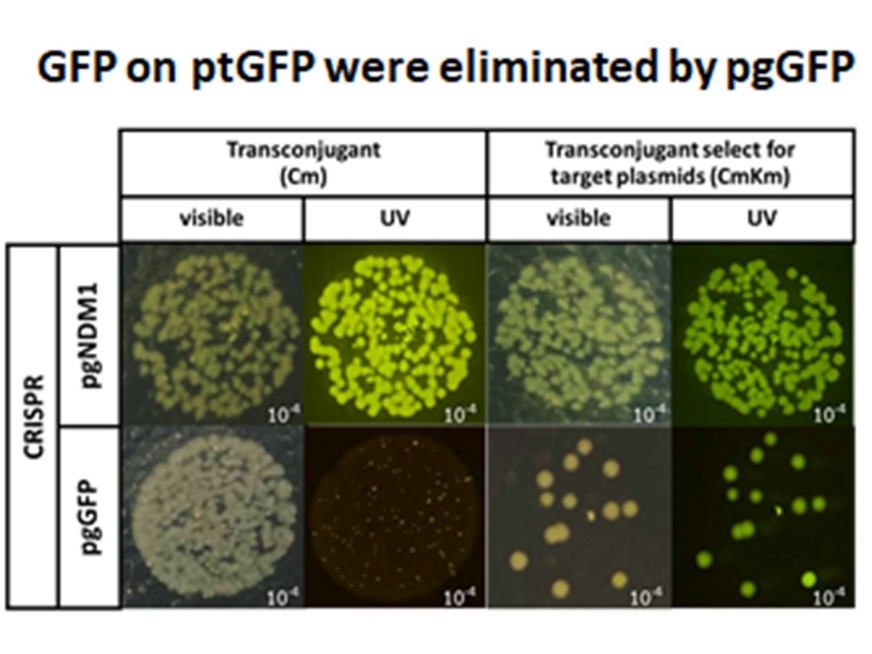


**Figure S2** Colony images of recipient cells with ptGFP after receiving pgGFP or pgNDM1 without or with selection for the target plasmid. The images were taken under visible light and UV light. pgGFP can knock out GFP or eliminate ptGFP, leading to the loss of fluorescent signal and kanamycin resistant marker (bottom row). pNDM1 had no observable effect on ptGFP (top row).


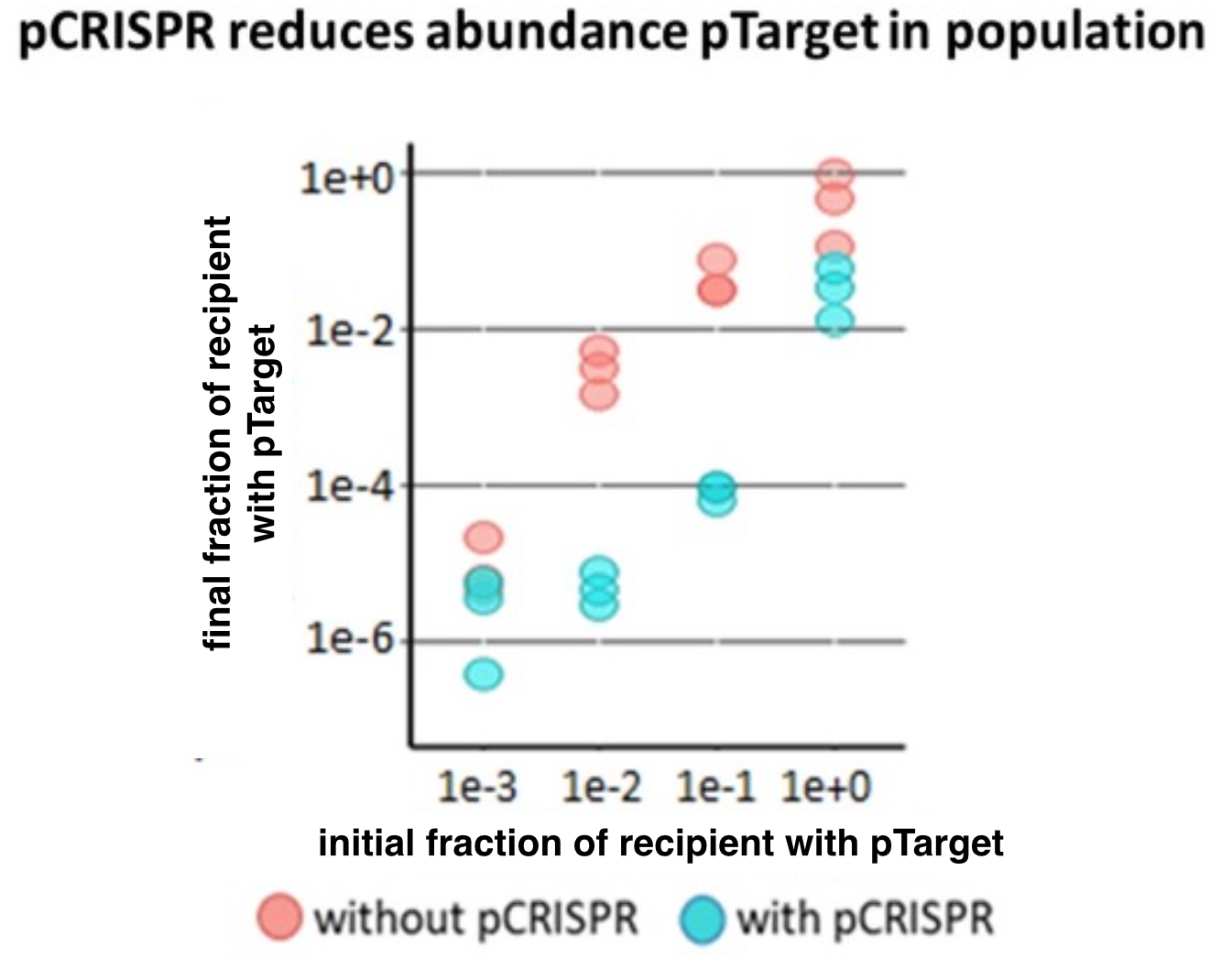


**Figure S3 pCRISPR reduced pTarget abundance in recipient population.** Donors with pCRISPR were mated with recipients at 1:180 ratio. At the beginning of the experiment, 0.1%, 1%, 10% or 100% of recipients had pTarget. The final ratio between recipients with pTarget and total recipients were measured after 18 h. of mating (no transconjugant selection).


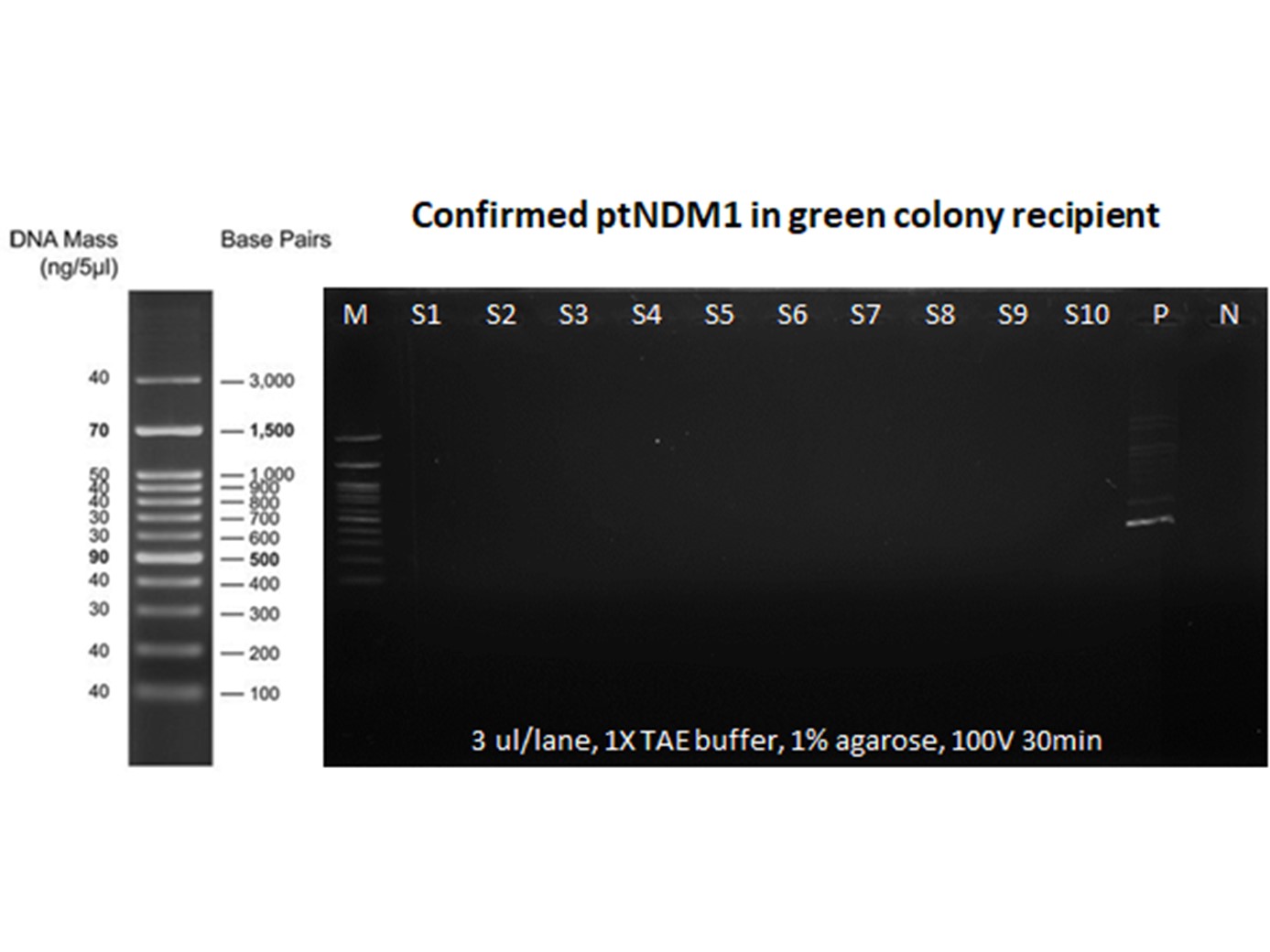


**Figure S4 PCR confirmation of total pTarget loss in recipient population.** Ten GFP expressing colonies (S1 – S10) were randomly chosen from LB (no selection) plate from Figure 4 experiment. The presence of pTarget was tested using colony PCR with a pair of primers specific to pTarget (expected product size 440 bp). Lane P and N show PCR results from positive (pTarget) and negative (SAR08 without plasmid) control. This result implies that none of recipient that had pSubst (expressing GFP) retained pTarget.


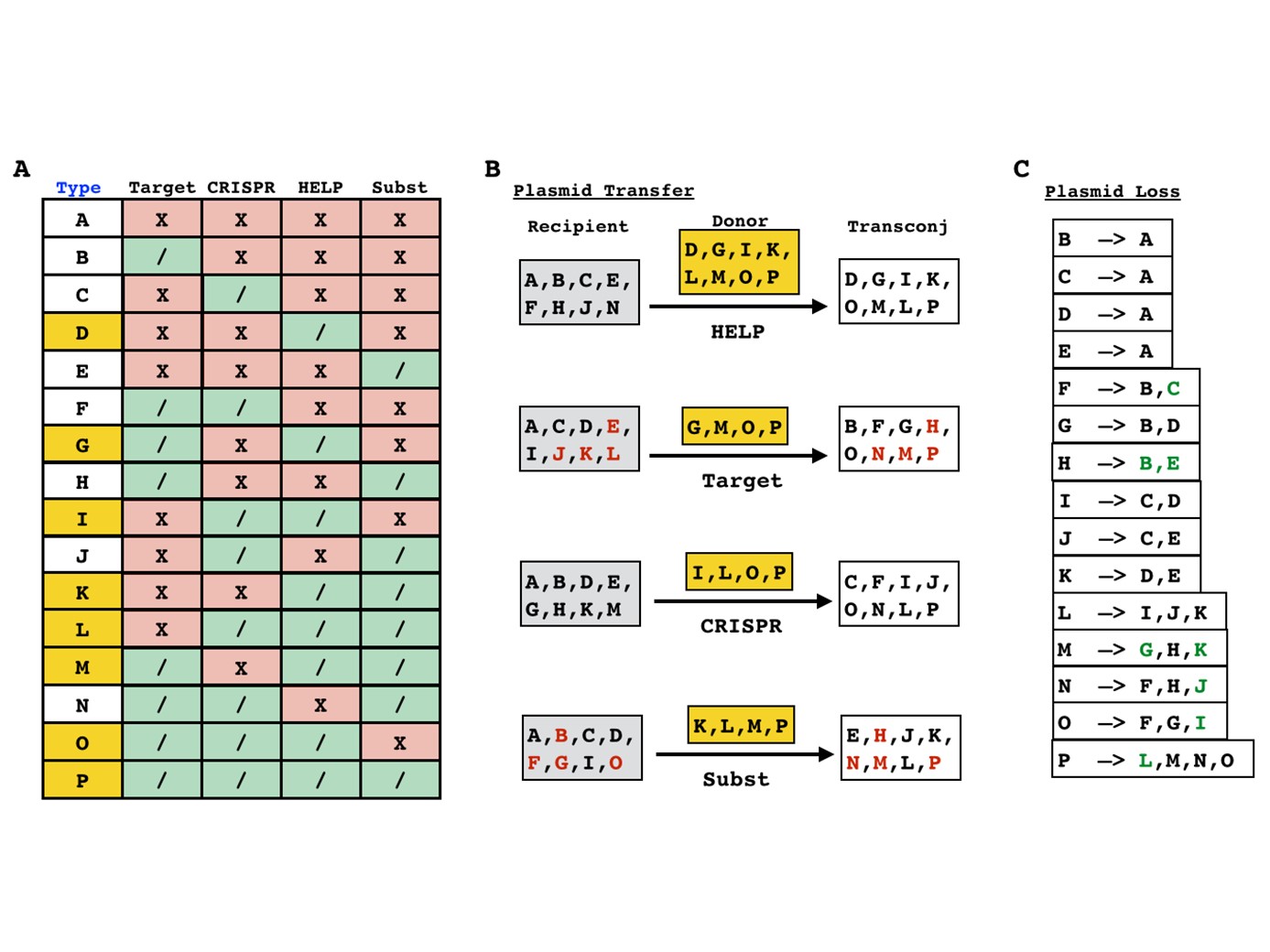


**Figure S5 Cell types and transitions between cell types in a four-plasmid population.** **(A)** A table showing all 16 possible cell types and plasmid(s) associated with each cell type. The first column (“type”) shows the name of all 16 cell types: A, B, C, …, O and P. The names of cells that have pHELP (and thus can function as a donor) are highlighted in yellow. The second to fifth column (“Target”, “CRISPR”, “HELP” and “Subst”) indicate whether each particular plasmid (pTarget, pCRISPR, pHELP and pSubst, respectively) is present (“/”) or absent (“X”) from each cell type. **(B)** A diagram showing all 120 possible transitions between cell types due to conjugative transfer of a plasmid. The names under the arrows indicate which plasmid is transferred; the lists of cell names in yellow boxes above the arrow indicate all possible donor cell types that can deliver each type of plasmid. The list of cell names in each grey box on the left of each arrow indicates all possible recipients for each type of plasmid. The list of cell names in each white box on the right of each arrow indicates corresponding transconjugants that would emerge after plasmid transfer. For example, cell type A and B can be recipients for pCRISPR (from cell type I, L, O or P) and turn into cell type C and F, respectively. Cell type names in red indicate conjugative transfer of a new plasmid from the same incompatibility group as existing plasmid in a recipient cell. This includes the transfer of pTarget into a recipient that already has pSubst or vice versa. Due to plasmid incompatibility, we set transfer rate constant to zero by default. (C) A diagram showing all 32 possible transitions between cell types due to plasmid loss. The left side of each arrow shows the original cell type; the right side of the arrow shows all possible cell new cell type after a single plasmid loss. Cell type names in green indicate that these cell types emerge due to CRISPR mediated plasmid loss (i.e., co-existence of pCRISPR and pTarget leads to loss of pTarget) or due to plasmid incompatibility (i.e., co-existence of pTarget and pSubst leads to the loss of either plasmid). By default, we set the rate constants of plasmid loss to zero except of those indicated in green.


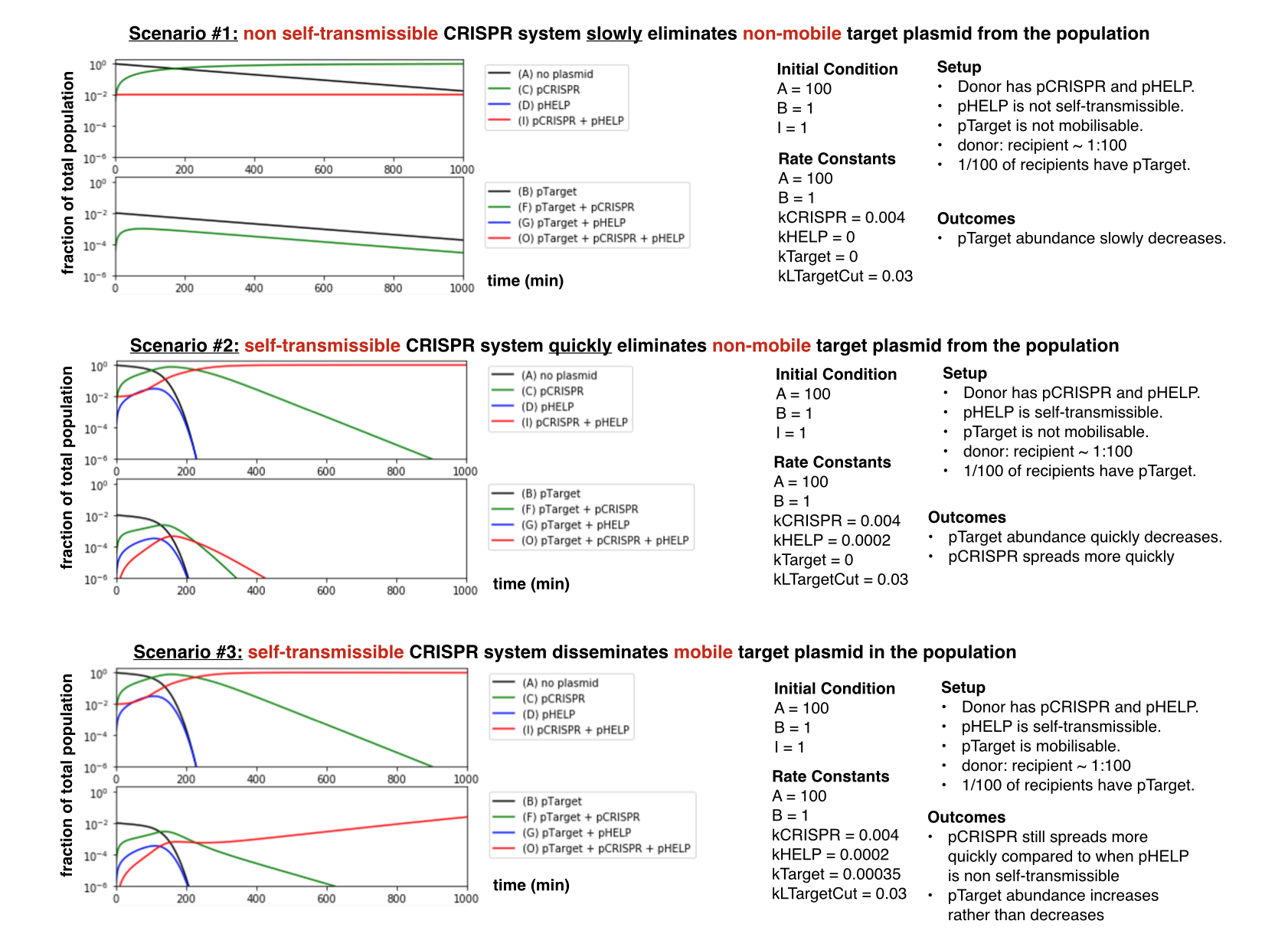


**Figure S6 Dissemination of CRISPR target plasmid by a self-transmissible CRISPR delivery system.** The figure shows three simulation results on the left and the parameter sets used in each simulation. The simulation results are presented as the changes in abundance of each cell type over time.


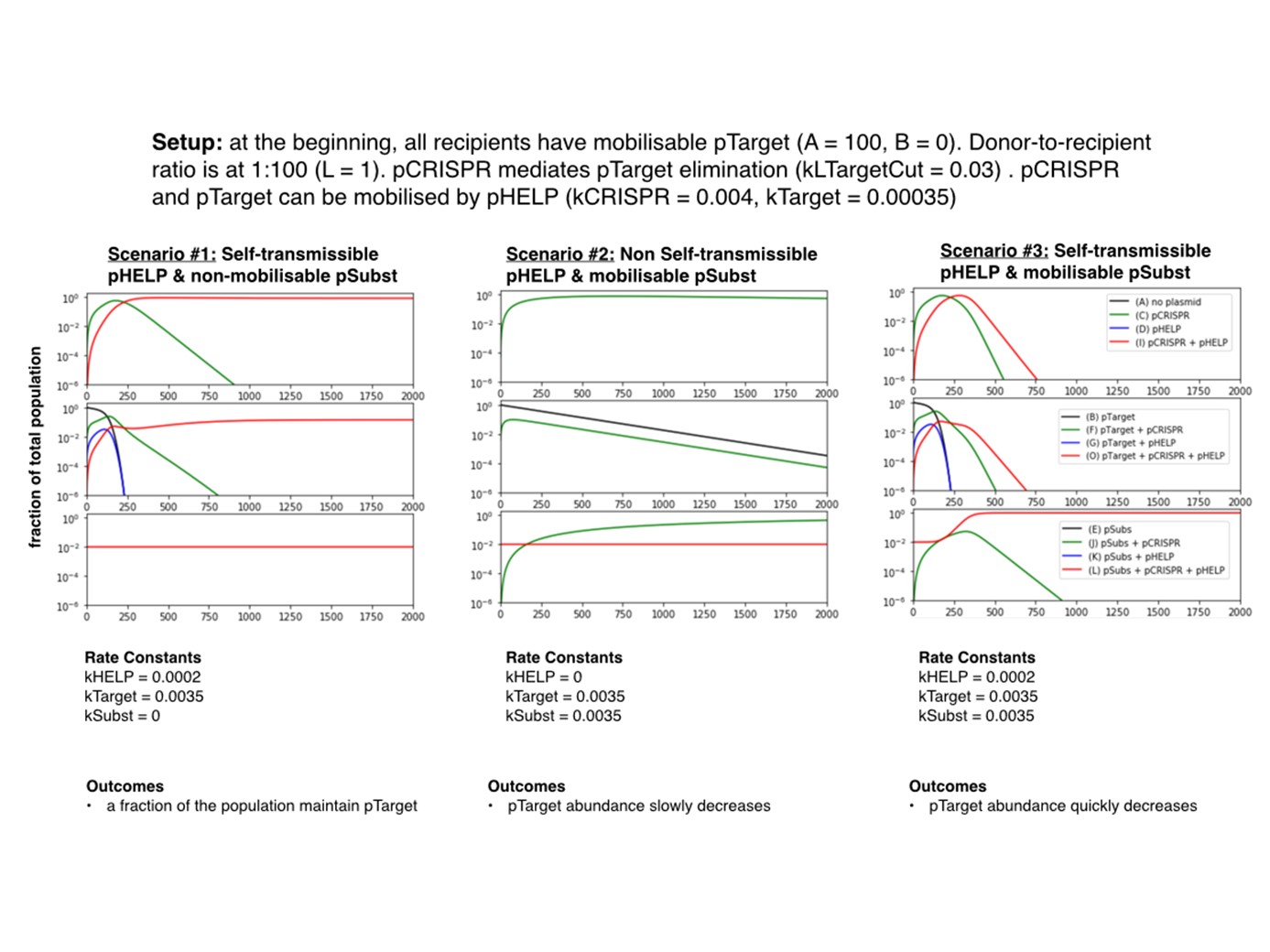


**Figure S7 The use of incompatible plasmid for elimination the target plasmid.** The figure shows three simulation results and the parameter sets used in each simulation. The simulation results are presented as the changes in abundance of each cell type over time.

**Kinetic Model Setup**

We created a mass-action kinetic deterministic model to recapitulate plasmid transfer dynamics observed in our experiment. For simplicity, we assumed that cell population was well-mixed and different cell types had approximately the same growth rate. The system consisted of four different plasmids: pTarget, pCRISPR, pHELP and pSubst. pHELP was the only conjugative self-transmissible plasmid. pTarget, pCRISPR and pSubst were mobilizable plasmid which can only be transferred from donor cells to recipient cells when pHELP also exist in the donor cells. pSubst and pTarget belong to the same plasmid incompatibility group and thus cannot co-exist stably in the same host cell. pCRISPR can express Cas9 and gRNA targeting a loci on pTarget. When pCRISPR and pTarget are in the same host cell, pTarget will be cleaved and eliminated.

With these four plasmids, there are 16 (2^4^) possible ‘cell types’ (A, B, C, …, O and P) classified by the presence or the absence of each plasmid (Figure S5, left). A cell can be converted from one type to another type when it loses its plasmid or receives a new plasmid (via conjugation). With 16 possible cell types, there are 240 (16 x 15) possible conjugative donor-recipient pairs. However, not all of these possible pairs can lead to a change in recipient cell type. The donor cell needs to have pHELP and the transferred plasmid need to be absence from the recipient cells (otherwise, there will not be any change of cell type) . We classify conjugative interactions into four classes based on the plasmid being transferred (Figure S5, right, below the arrows). A possible donor cell for each class of conjugative interaction is a cell that has plasmid to be transferred as well as a helper plasmid (Figure S5, right, yellow box). For instance, cell type I, L, O and P are all possible donor cells for transferring pCRISPR because all these cell types have both pCRISPR and pHELP. A possible recipient cell for each class of conjugative interaction is a cell that does not have the plasmid being transferred (Figure S5, right, grey boxes). For instance, cell type A, B, D, E, G, H, K and M are all possible recipients of pCRISPR because pCRISPR is absence from these cell types. Once the recipient cell received a plasmid, it will be converted to another cell type. For instance, a cell type A that receives pCRISPR (from cell type I, L, O or P) will become cell type C; a cell type B that receives pCRISPR (from cell type I, L, O or P) will become cell type F.

There are 8 possible pHELP donors and 8 possible pHELP recipients amounting to 8 x 8 = 64 possible pHELP transfer interactions. There are 4 possible donors and 8 possible recipients for pTarget, pCRISPR or pSubst, amounting to 4 x 8 = 32 possible pTarget, pCRISPR or pSubst transfer interactions. Overall, there are 64 + 32 + 32 + 32 = 160 different plasmid transfer interactions that lead conversion of one cell type to another. A cell of one type may lose its plasmid(s) and become another cell type. For instance, a cell type I may lose its pCRISPR or pHELP and thus become a cell type D or a cell type C, respectively. Of our 16 possible cell types, there are 32 possible plasmid loses. To simplify the model, we assumed that there was no plasmid loss except for the following scenarios. First, when pCRISPR and pTarget are in the same cell, pTarget will be eliminated due to CRISPR/Cas mediated plasmid cleavage. Second, when pTarget and pSubst are in the same cell, either plasmid could be lost due to plasmid incompatibility.

Kinetic models were simulated using Tellurium, a Python environment for reproducible dynamical modeling of biological networks (<http://tellurium.analogmachine.org/>). See the supplementary iPython notebook file for an example of programming code used for generating simulation results.

**Simulation: Self-transmissibility of CRISPR delivery systems may hinder target elimination.**

Self-transmissibility of CRISPR delivery systems helps spread CRISPR antimicrobial more quickly in microbial population compared to a system in which CRISPR can only be delivered directly from the original conjugative donor cell. However, self-transmissible delivery systems may also mobilize undesirable gene(s) including CRISPR target we intend to eliminate. Thus, self-transmissible delivery system may, under some condition, slow down or even reverse the effects of CRISPR antimicrobials.

Here we simulate three plausible scenarios related to the delivery of self-transmissibility of CRISPR delivery system and the spread or elimination of CRISPR target (Figure S6). In all scenarios, we have low ~ 1:100 donor-to-recipient ratio and only 1% of recipient population initially had pTarget (similar to the setup we had in Figure 2E experiment). In scenario #1, CRISPR delivery system is not self-transmissible and the CRISPR target is not mobilizable. Specifically, the conjugation rate of pHELP and the mobilization rate of pTarget are set to zero. This scenario is equivalent of having conjugative machinery stably integrated in the genome of original donor cell (such as EcGT2 in our experiment). Thus, pCRISPR can only be mobilized directly from the original donor cell and not from transconjugant. Under our chosen kinetic parameter set, pCRISPR slowly spread throughout the population while the percentage of cells with pTarget gradually decreases over time. Note that in non-well mixed population such as in our experiment or biofilm, pCRISPR is unlikely to reach the majority of the population as all recipients need to come into direct contact with the original donors. When donor-to-recipient ratio is low (at least 1:100 in this simulation and in our experiment), the majority of recipient will not receive pCRISPR and thus pTarget cannot be completely eliminated. Such limitation is one of the key motivations for having self-transmissible delivery systems whether on the same vector as pCRISPR (like in Hamilton et al 2019) or on a separated helper vector (like in Routsalainen et al 2019 or in our work).

In scenario #2, the CRISPR delivery system is self-transmissible but the CRISPR target is still not mobilizable. Specifically, the conjugation rate of pHELP is positive while the mobilization rate of pTarget is still at zero. This scenario is equivalent of having conjugation machinery on a conjugative plasmid (i.e., pHELP) while the CRISPR target is on the genome or non-mobilizable plasmid. Using similar kinetic parameter set as in scenario # 1 (but with positive pHELP conjugation rate), pCRISPR spreads throughout the population and pTarget is eliminated from the population significantly more quickly than in scenario #1. Thus, this simulation result highlights the benefit of having self-transmissible delivery systems. Moreover, in non well-mixed population and at low donor-to-recipient ration, having self-transmissible delivery is likely to be the only way to deliver pCRISPR to the entire population and completely eliminate pTarget.

In scenario #3, the CRISPR delivery system is self-transmissible and the CRISPR target is mobilizable. In other words, both conjugation rate of pHELP and mobilization rate of pTarget are positive. This scenario is similar to our experiment (Figure 2E). Using similar kinetic parameter set as in scenario # 2 (but also with positive pTarget mobilization rate), pCRISPR still spreads quickly throughout the population (as in scenario #2) but the abundance of pTarget increases rather than decreases. Notice that the majority of pTarget resides within cell type O (which has pTarget, pCRISPR and pHELP). This is because while pCRISPR can mediate the elimination of pTarget, more pTarget plasmids are being added into the population due to the presence of pHELP. Thus, this simulation shows that the benefit of having CRISPR antimicrobial spread quickly by self-transmissible delivery system could be negated by the cost of having the CRISPR target being disseminated as well.

**Simulation: Incompatible plasmid delivery may speed up target plasmid elimination**

Plasmids from the same incompatibility group (Inc) cannot co-exist stably inside the same host cell. When one plasmid already exists inside the cell, the other plasmid from the same Inc is unlikely to be able to enter, compete with and replace the existing one. Thus, we reasoned that we could use plasmid incompatibility to mediate irreversible plasmid replacement that speed up the process of target plasmid elimination.

In the following simulations, we assume a population of bacteria in which every cell has a CRISPR target plasmid we like to eliminate (B = 100%). The donor cell has three plasmids: pCRISPR, pHELP and pSubst. Initial donor-to-recipient ratio is at 1:100. When pTarget is in the cell, it prevents the establishment of pSubst and vice vera. pCRISPR can only eliminate pTarget and not pSubst. For scenario #1, pHELP is conjugative self-transmissible while pTarget and pCRISPR are mobilizable (similar to Figure S6, bottom). pSubst exists in the initial donor population but cannot be mobilized and thus has no effect on pTarget in the population. Here, the spread of pCRISPR, accelerated by self-transmissible pHELP, led to the reduction of pTarget in the population (Figure S7, left). However, the abundance of pTarget reaches a steady-state at approximately 10% of the population. Similar to an example above (Figure S6), pHELP mediates the dissemination of pTarget thereby negating the effect of pCRISPR. For scenario #2, pSubst is mobilizable but pHELP is not self-transmissible (i.e., conjugation machinery in the genome). This scenario is similar to that of experiment shown in Figure 3. pCRISPR is delivered slowly throughout the population and, in the process, eliminating pTarget and allowing pSubst to spread. According to this simulation, all pTarget in the population could be eliminated and replaced by pSubst. Nonetheless, as discussed above, in non-well mix population with low donor-to-recipient ratio, the majority of recipients would never come into direct contact with the initial donors. Thus, most pTarget will remain in the population.

In scenario #3, pHELP is self-transmissible while both pCRISPR and pSubst are mobilizable. According to this simulation, pCRISPR quickly spread throughout the population and eliminate pTarget. pSubst quickly replaces and prevent further dissemination of pTarget. Thus, pTarget is completely eliminated from the population. Similar to Figure 4C of the main text, this simulation shows that the combination of self-transmissible CRISPR delivery and the use of plasmid incompatibility replacement allow us to completely eliminate a mobilizable target plasmid.

**Generalizability of a plasmid elimination and substitution system**

To demonstrate generalizability of a plasmid substitution system, we performed an experiment similar to that shown in Figure 3 but using a different pair of target and substitution plasmids (Figure S8). pTarget-RK2 and pSubst-RK2 function as target plasmids and substitution plasmids, respectively. These two plasmids have RK2 origin of replication, rather than pBBR1 origin of replication (as in pTarget and pSubst). pTarget-RK2 has a fragment of NDM1 gene sequence which can be cleaved by CRISPR/Cas9 from pCRISPR. pSubst-RK2, on the other hand, has no NDM1 sequence and thus cannot be cleaved by CRISPR/Cas9 from pCRISPR.

We conjugated donor cells harboring pCRISPR and pSubst-RK2 to recipient cells harboring pTarget-RK2. After the first round of conjugation with pCRISPR and pEMPTY selection, all transconjugants (Figure S8 ) receiving pCRISPR lose pTarget-RK2. After the second round of conjugation selecting for pTarget-RK2 or pSubst-RK2, the majority of tested transconjugants had pSubst-RK2. In a negative control experiment, we conjugated donor cells pEMPTY and pSubst-RK2 to recipient cells harboring pTarget-RK2. After two rounds of conjugations, all tested transconjugants had both pSubst-RK2 and pTarget-RK2.

We concluded that pCRISPR can mediated the substitution of pTarget-RK2 in the recipient cells by pSubst-RK2. Target elimination and replacement process appeared to be slower than when we used pSubst to replace pTarget (Figure 3). Here, we needed two rounds of conjugation in order to have pSubst-RK2 to spread to the majority of population. Nonetheless, pTarget-RK2 and pSubst-RK2 can co-exist in the same cell, at least temporarily.


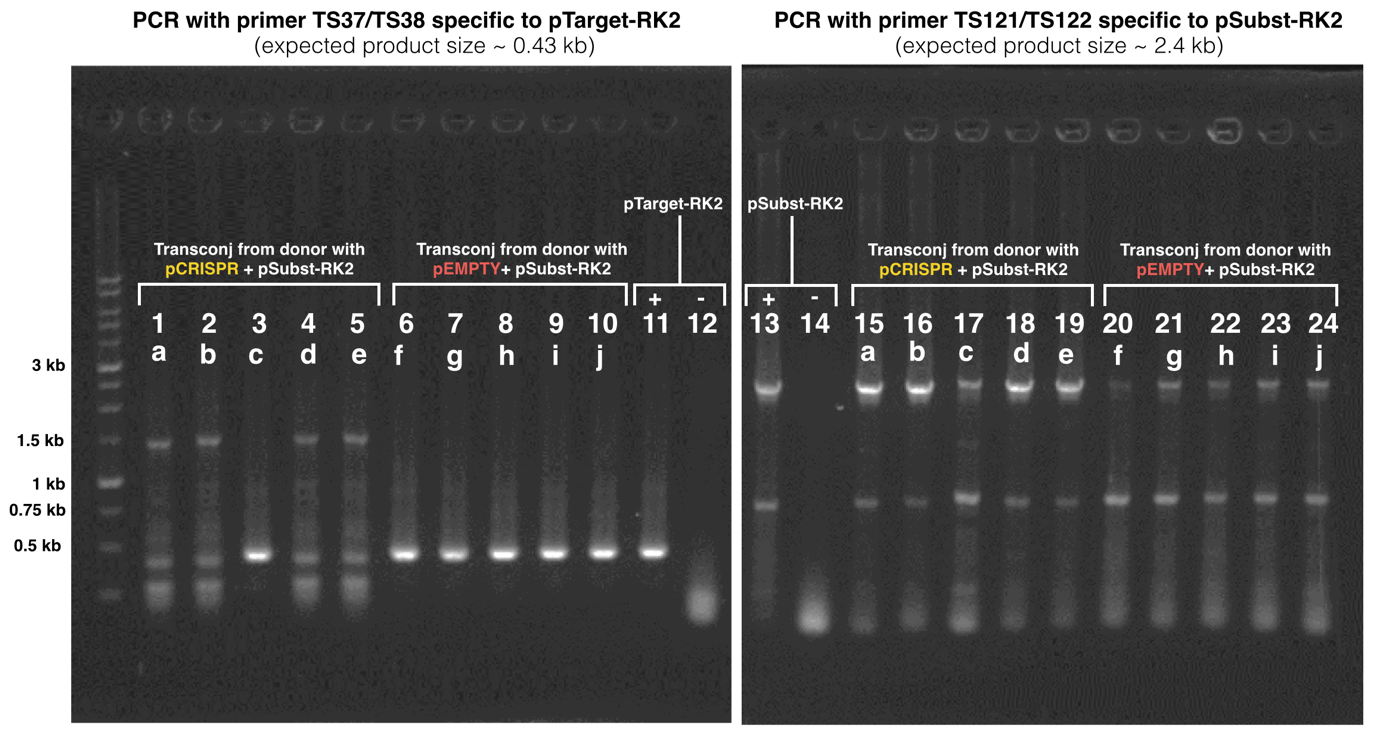


**Figure S8 CRISPR mediated elimination and substitution of RK2 mobilisable plasmid.** Agarose gel electrophoresis images show colony PCR results using primer TS37/TS38 specific to pTarget-RK2 (left) and primer TS121/TS122 specific to pSubst-RK2 (right). Lane 11-12 and 13-14, respectively, show positive and negative PCR results from sample with or without pTarget-RK2 and pSubst-RK2. Lane 1-5 and 15-19 show colony PCR results using 5 different transconjugant colonies (a-e) derived from two rounds of conjugation using EcGT2/pCRISPR/pSubst-RK2 as donor and SAR08/pTarget-RK2 as recipient. Lane 6-10 and 20-24 show colony PCR results using 5 different transconjugant colonies (f-j) derived from two rounds of conjugation using EcGT2/pEMPTY/pSubst-RK2 as donor and SAR08/pTarget-RK2 as recipient.

We also performed an additional experiment to test whether the presence of pSubst could hinder the re-establishment of pTarget in the recipient population. Here we used EcGT2/pEMPTY/pTarget as donors and SAR08 (with or without pSubst) as recipients. Donors and recipients were conjugated as described in main text material and methods at 1:1 donor-to-recipient ratio for 18 hr. After conjugation, the donor-recipient mixes were plated on LB agar with chloramphenicol to select for transconjugants that received pEMPTY. After an overnight incubation at 37 C, 26 colonies from each conjugation experiment were streaked on LB agar with kanamycin to test for the presence of pTarget or pSubst. After an overnight incubation, six colonies that grew on LB with kanamycin were chosen randomly and colony PCR to test for the presence of pEMPTY, pTarget, and pSubst.

For an experiment using SAR08 (without pSubst) as recipients, 12 out of 26 colonies from an LB chloramphenicol plate can grow on an LB kanamycin plate. All six randomly chosen colonies were confirmed to have pEMPTY and pTarget. For an experiment using SAR08/pSubst as recipient, all colonies from an LB chloramphenicol plate can grow an LB kanamycin plate and expressing GFP. Only two out for six randomly chosen colonies were confirmed to have pTarget. Interestingly, these two colonies also had pSubst.

From these experiments, we conclude that the presence of pSubst in a recipient cell does not completely prevent the entry and re-establishment of pTarget in the population. However, all tested recipient without pSubst received pTarget while only a third of tested recipient with pSubst received pTarget. This implies that the presence of pSubst might partially interfere with the spread of pTarget in recipient population. Such interference could be a combination of competition between incompatible plasmids and the absence of selective advantage of transconjugant receiving pTarget. Future experiment should investigate the extent to which one plasmid can replace another plasmid of the same incompatibility group for different types of plasmids and conjugative delivery mechanism.


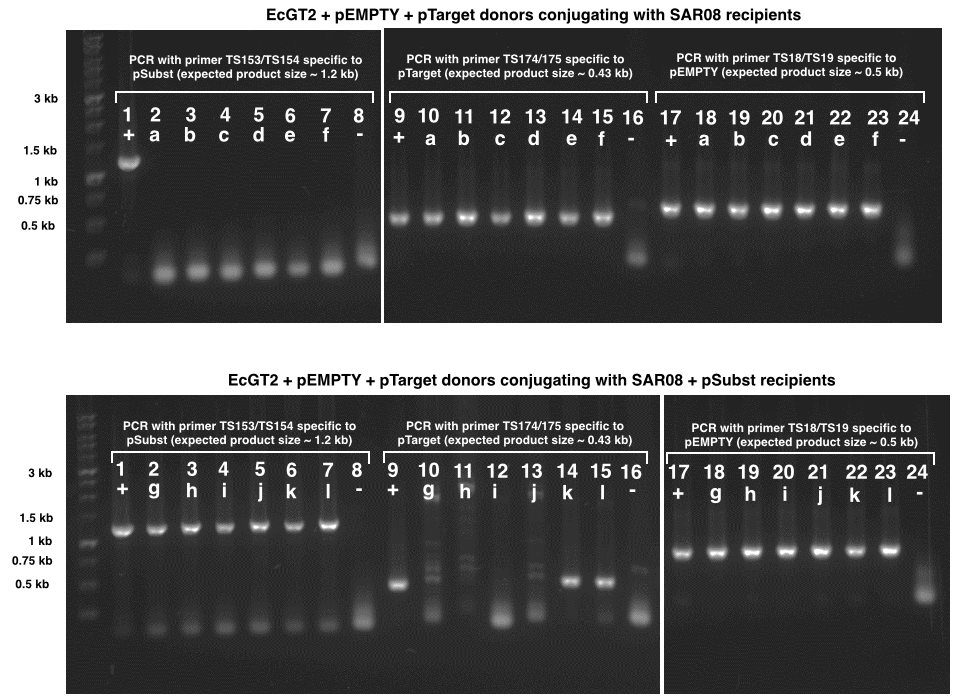


**Figure S9 pSubst in recipients hindering pTarget entry.** Agarose gel electrophoresis images show colony PCR results from conjugation experiment between EcGT2/pEMPTY/pTarget donors and SAR08 recipient (top) or SAR08/pSubst (bottom). Primers for colony PCR were specific to pSubst (lane 1-8), pTarget (lane 9-16) or pEMPTY (lane 17-24). ‘+’ and ‘-‘ indicate PCR results of positive and negative controls. Letter a – l indicate PCR results from different colonies.
